# Bio-Based Adsorption as Ecofriendly Method for Wastewater Decontamination: A Review

**DOI:** 10.3390/toxics11050404

**Published:** 2023-04-24

**Authors:** Juliette Vievard, Abdellah Alem, Anne Pantet, Nasre-Dine Ahfir, Mónica Gisel Arellano-Sánchez, Christine Devouge-Boyer, Mélanie Mignot

**Affiliations:** 1 University Le Havre Normandie, UNIHAVRE, UMR 6294 CNRS, LOMC, 76600 Le Havre, France; juliette.vievard@insa-rouen.fr (J.V.); abdellah.alem@univ-lehavre.fr (A.A.); anne.pantet@univ-lehavre.fr (A.P.); nasre-dine.ahfir@univ-lehavre.fr (N.-D.A.); 2 University Rouen Normandie, UNIROUEN, COBRA UMR CNRS 6014, INSA, Avenue de l’Université, 76800 Saint-Etienne-du-Rouvray, France; marellano.san@gmail.com (M.G.A.-S.); christine.devouge-boyer@insa-rouen.fr (C.D.-B.)

**Keywords:** adsorption, biomass, water treatment, pollutant, isotherm, kinetic studies

## Abstract

Intense human activities have for years contributed to the pollution of the environment by many dangerous pollutants such as heavy metals, pesticides, or polycyclic aromatic hydrocarbons. There are many conventional methods used to control pollution, with practical and/or financial drawbacks. Therefore, in recent years, an innovative, easy-to-implement and inexpensive adsorption method has been developed to recover waste and clean up water from micropollutants. Firstly, this article aims to summarize the issues related to water remediation and to understand the advantages and disadvantages of the methods classically used to purify water. In particular, this review aims to provide a recent update of the bio-based adsorbents and their use. Differently from the majority of the reviews related to wastewater treatment, in this article several classes of pollutants are considered. Then, a discussion about the adsorption process and interactions involved is provided. Finally, perspectives are suggested about the future work to be done in this field.

## 1. Introduction

Population growth has generated greater requirements, which continually leads to increased industrial, agricultural, and domestic activities and, therefore, pollution of water and environmental ecosystems [1]. Water pollution has become a public issue, and the main cause appears to be anthropogenic. Two types of pollution sources have been identified: point source (a directly identifiable source that affects mainly the nearby area, such as the release of fuel from a tanker) and non-point source (a hardly identifiable source from which pollutants are delivered by different origins, such as rainwater runoff on road areas) [2,3]. Both groundwater and surface water can be polluted, and the degradation of the one can lead to the degradation of the other [2].

Different types of pollutants can be found in water, such as inorganic pollutants (nutrients, halogens, and heavy metals), organic pollutants (pesticides, dyes, polycyclic aromatic hydrocarbons, and pharmaceutical and personal care products), microbial contaminants, and radioactive and thermal pollutants [3]. The presence of these pollutants in water at continuously increasing concentrations has begun to have negative effects on human health, wildlife, and plants. For example, the presence of dyes in waters has affected the photosynthesis process by the inhibition of sunlight transmission, which has disrupted the food chain of aquatic life [4].

Many methods can be used to remove pollutants from water: biological treatment, chemical precipitation, ion exchange, membrane process, chemical oxidation or reduction, coagulation or flocculation, reverse osmosis, and adsorption [5,6,7,8]. Some of these methods have been reported to be effective for the removal of pollutants, but they present some disadvantages as well. For example, the ion exchange method involves the renewal of expensive ion exchange resins, and the chemical reduction and precipitation methods can result in the production of hazardous by-products, such as nitrite and ammonia for nitrate removal by the first cited method [9]. The use of the adsorption method is currently under investigation, and especially bio-based adsorption. It is an adsorption process aiming at removing or recovering organic and inorganic substances in aqueous solutions using bio-based materials. It has many advantages and can be considered as one of the most effective and economical methods for pollutants removal [10]. Moreover, pollutants can be removed at low concentrations in a safe and easy way, and adsorbents can be used in both batch and column experiments [11].

This article provides an overview of wastewater pollutants and their effects on human health and environment. Classical removal techniques of pollutants and the effects of the different parameters involved in adsorption are described (temperature, pH, contact time, initial pollutant and adsorbent concentrations, and competition with other pollutants). Next, the interaction between pollutants and biomass and the techniques for their determination are presented (kinetic and isotherm models). In addition, different types of bio-based adsorbents that have already been reported in literature are also presented for the removal of pollutants from waters. Finally, the future outlook for polluted water treatment techniques and the related issues are also discussed in this work.

## 2. Source of Water Pollution

**Figure 1:**
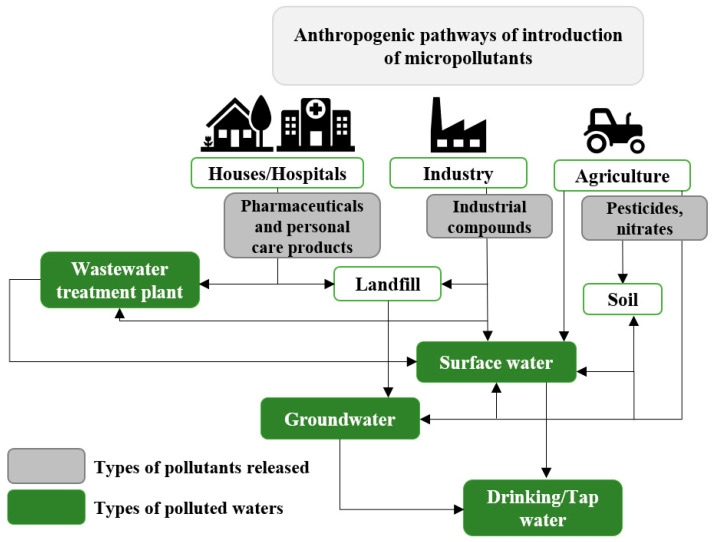
Pathways of introduction of micropollutants into the environment. Modified from Ref. [31].

Environmental pollution is mainly due to anthropogenic reasons, such as the intensive production of household necessities or dense traffic generating toxic emissions, as shown in Figure 1 [12]. This figure presents the emission sources of different classes of pollutant as well as the types of water that may be affected.

Surface waters, such as lakes or rivers, can be contaminated either directly by pollution sources or by urban stormwater when it is discharged into them [13]. Indeed, due to the ever-increasing impermeability of the soil, runoff water can no longer infiltrate naturally; instead, it runs off and is loaded with micropollutants that accumulate at the surface during dry weather [14]. In cities with a separate sewer system, water is released directly into surface waters without treatment. Contrarily, for cities with a combined sewer system, water is treated in a wastewater treatment plant. However, in case of heavy rainfall, volumes exceed the treatment plant capacity, so water is released without specific treatment through storm overflows or through alternative techniques such as retention ponds [15].

Industrial activities are partly responsible for water contamination. First, it has been shown that wastewater from electroplating plants may contain nitrates used in the pickling process, or heavy metals used for coatings [16]. Heavy metals can also be discharged into water from other types of industry: petroleum processing, metallurgy, chemicals, or power stations [16,17]. Second, textile, paper production, leather tanning, food, and pharmaceutical industries can be cited as the origin of the occurrence of persistent dyes in industrial wastewaters, usually used as a coloring agent [18,19,20]. Third, polycyclic aromatic hydrocarbons (PAHs) can be produced by natural and anthropogenic activities [21]. The latter type includes personal activities such as vehicle emissions, but also incomplete combustion of fossil fuels or biomass by industries [22,23]. Moreover, substantial levels of polychlorinated biphenyls (PCBs) can be found in the global ecosystem due to their extensive use between the 1930s and 1970s as commercial products [24]. Dioxins are produced unintentionally as by-products of the combustion of organic materials in the presence of chlorine [25]. Finally, pharmaceutical compounds are emanated into the aquatic environment through industrial manufacturing waste, human and animal excrement, or hospital wastewaters [26]. These pollutants are particularly found in waters of the most developed countries, where medicines are consumed daily by many people.

It has been established that agriculture also contributes significantly to the contamination of water. In particular, the intense use of pesticides (herbicides, fungicides, and insecticides) required to intensify the food production to support a growing population leads to an increase of their presence in water. During rain events, they are transported over long distances and driven to water sources [27]. Additionally, the massive use of nitrogen fertilizers has led to an increase of nitrates in soils. They were released into groundwater because of their high solubility and repulsive force with soil, especially with soil particles and soil organic matter [28,29,30].

## 3. Toxic Effects of Pollutants

### 3.1. Nitrates

The harmful effects of pollutants on human health and environment have been documented many times. It turns out that a moderate concentration of nitrates favors the growth of organisms. However, excessive amount of nitrates can lead to an increased growth of organisms such as algae, and finally to eutrophication [30]. Additionally, by reducing the ability of red blood cells to carry oxygen, contamination of drinking water with high concentrations of nitrates can cause blue baby syndrome [32].

### 3.2. Dyes

The absorption and reflection of sunlight in water can be physically stopped by dyes because of their aromatic structure, which inhibit its penetration into the photic zone of the aquatic environment and reduce the natural phenomenon of photosynthesis [33]. Synthetic dyes can be categorized as carcinogenic and mutagenic compounds to both humans and animals. Cationic dyes in particular can be classified as toxic compounds as they can reach the entire food chain [5,34] due to their high stability and inability to degrade. Health problems such as irritation to skin and eyes, nausea, vomiting, or more serious symptoms such as respiratory tract problem have been identified [5,35].

### 3.3. PAHs, PCBs and Dioxins

Due to their structure containing two or more rings, PAHs are persistent in the environment. Consequently, they travel over a long distance, which can lead to their bioaccumulation [36]. They are well known for their toxicity in human health due to their mutagenic and carcinogenic properties [10]. As a matter of fact, having the ability to bind to DNA, disordered effects leading to tumor initiation can be caused by PAHs [37,38].

Some of the physicochemical properties of PCBs (low vapor pressure and low aqueous solubility) lead to their massive persistence in the environment, and thus to their bioaccumulation. They can consequently be found in the food chain, especially in fish and aquatic species [24]. PCBs and dioxins have negative effects on the environment and human health. They can cause skin toxicity, immunotoxicity, neurotoxicity, adverse reproductive effects, teratogenicity, endocrine disruption, and cancer susceptibility [25].

### 3.4. Heavy Metals

Similarly, heavy metals can be regarded as one of the most toxic families of pollutants because of their high solubility in the aquatic environment and their persistence, leading to their bioaccumulation [39]. They can be responsible for chronic or acute diseases [40]. For example, unusual and excessive exposure to nickel can have negative effects on the respiratory system, which may result in nasal lung cancer or asthma [41,42].

### 3.5. Pharmaceutical and Personal Care Products

Due to their chemical stability and, therefore, persistence, pharmaceutical compounds are found in all types of waters: groundwater, surface water, and drinking water. Their presence can generate a harmful exposure of aquatic organisms, which may cause a resistance of micro-organisms to drugs, an endocrine disruption, and thus a decline in biodiversity [43].

### 3.6. Pesticides

Pesticides are extremely dangerous for human beings because they have a carcinogenic, mutagenic, and teratogenic potential. Indeed, they can be responsible for DNA mutations leading to chromosomal changes, cancers, malformations, or infertility. In addition, they have a tragic effect on non-target species because they are easily sprayed and can move from their original deposition site [44,45].

## 4. Conventional Methods of Water Treatment

**Figure 2:**
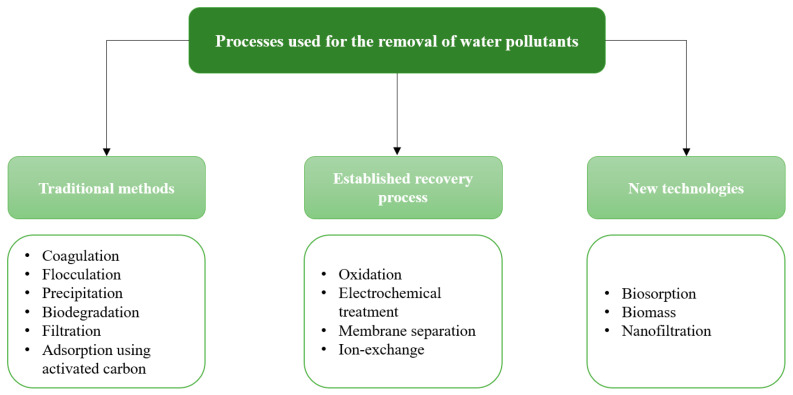
Conventional methods of water treatment and examples. Modified from Ref. [52].

Over the last few decades, water depollution has become a major issue for researchers. For this purpose, many physicochemical and biological methods (listed in Table 1) have been developed to clean up water from different types of micropollutants: biological treatment, chemical precipitation, ion exchange, membrane process, chemical oxidation or reduction, coagulation or flocculation, reverse osmosis, electrochemical treatment, and adsorption, as summarized in Figure 2 [5,6,7,8]. 

Membrane process is a physical method that has been used in different water treatment studies. For wastewater treatment, water is pumped under pressure through a semi-permeable membrane. Depending on the membrane pore size, different types of pollutants can be retained. For example, zinc-doped aluminum oxide nanoparticles/polysulfone mixed matrix membranes have been developed by Sherugar et al. (2021) to remove heavy metals from water [46]. Their work concluded that arsenic and lead were removed with efficiencies up to 87% and 98%, respectively. Reverse osmosis has a similar principle and can also be a useful method for water purification. In fact, Couto et al. (2020) demonstrated that reverse osmosis and nanofiltration were viable alternatives for removing pharmaceutical compounds from drinking water [47]. These methods offer high removal efficiency, no secondary pollution, and low energy consumption. However, good efficiency is highly dependent on the membrane used, including pore size and composition [48].

As far as chemical precipitation is concerned, it is one of the most common methods used in industry to remove different types of pollutant from water, such as heavy metals. This is justified by the low cost of implementation, its effectiveness in a wide range of temperatures, and its low operating cost and simplicity of process control [49]. The principle of this method is based on the formation of a separable solid substance from a solution, either by transforming the substance into an insoluble form or by changing the composition of the solvent to decrease the solubility of the substance, leading to precipitation. The chemical precipitation method has been used to remove copper, zinc, and lead from aqueous solutions using lime, soda, and sodium sulfide precipitants by Chen et al. (2018), the latter being the most efficient (removal rate of over 99.75%) [49]. However, chemical precipitation has the disadvantage of potentially producing secondary pollution [48]. Similarly, the coagulation–flocculation method can be used for the removal of suspended matter (sand, silt, etc.), colloidal matter (fine clays, bacteria, macromolecules, etc.), and dissolved matter [50]. 

Furthermore, ion exchange resins can also be employed in water treatment. These are insoluble granular substances with a macroporous structure containing anions or cations. They have the capacity to carry out a reversible stoichiometric chemical reaction, i.e., an exchange of their ions against those of the same charge present in water, without modification or physical alteration. This method is widely employed because of its simplicity, effectiveness, selectivity, and moderately low cost. For example, Kalaruban et al. (2016) have used a Dowex 21K XLT anion exchange resin (Dow Chemical Pte Ltd., USA) modified with iron incorporation for the removal of nitrates [51]. The Langmuir model was used to determine the adsorption capacity of the modified resin under imposed conditions (an ionic strength of 1 × 10^−3^ M NaCl and pH 6.5), this was 75.3 mg/g. The limitations of this method are a strong dependence on the structure of the resin and the environment of the solution [48,51].

Oxidation–reduction can also be applied in the water treatment cycle. The objectives can be variable: disinfection before domestic or industrial use to avoid any risk of bacteriological contamination, precipitation of dissolved compounds (iron, manganese, sulfides), or degradation of organic compounds that contribute to the chemical oxygen demand of water [52]. It can also be used to remove some hazardous pollutants from wastewater. For example, Antošová et al. (2020) worked on the use of ferrates to degrade PAHs in solution. Ferrates, due to their high oxidation potential, are considered as good chemical agents for water treatment. The study showed that ferrates can degrade PAHs, but the resistance to this degradation increases with their molecular weight. In fact, PAHs with two or three benzene rings were totally degraded within 30 min, while those with five or more rings were only partially degraded [53]. Concerning this method, the main disadvantage is the use of chemicals [52].

Among the electrochemical treatments, various methods are available for wastewater treatment, such as electrodeposition, electrochemical oxidation, electrochemical reduction, electroflocculation, electrocoagulation, etc. The last-mentioned method is considered to be the most effective. The principle is the production of destabilizing agents from sacrificial anodes for the removal of pollutants [52]. For example, the results obtained by Sharma et al. (2019) confirmed the effectiveness of this method, which removed over 90% of Cr(VI) and Pb(II) from polluted waters [54]. The main advantage of electrochemical treatment is the use of electrons in the process, which is environmentally friendly. Other advantages of this process are its flexibility, the possibility of automation, and the limited costs [48].

Another way to treat water is to use biological processes. Biological treatment by activated sludge is an important step in water treatment. In this process, micro-organisms transform the dissolved pollution into biological sludge by alternating aeration and resting phases in tanks. Biological treatment is the transformation of pollution by bacteria into carbon dioxide, treated water, and sludge. For treatment beyond wastewater treatment plants, studies are being conducted to clean up hazardous micropollutants, such as PAHs and dyes [46,55]. This type of treatment of organic contaminants is simple to implement and economically interesting. However, it is necessary to establish a favorable environment for the development of bacteria [52].

**Table 1:** Conventional methods of water treatment for different pollutants with their advantages and disadvantages.

Treatment Methods	Pollutant	Sample	Advantages	Disadvantages	References
Nanofiltration membrane	Dyes	Textile effluent	Effectiveness, no secondary pollution, and low energy consumption	Dependence on the membrane used	Panda et De (2015) [56]
Mixed matrix membranes	Heavy metals	Water			Sherugar et al. (2021) [46]
Nanofiltration membrane and reverse osmosis	Pharmaceutical compounds	Surface water			Couto et al. (2020) [47]
Chemical precipitation	Heavy metals	Water	Effectiveness, low operating cost, and simplicity	Secondary pollution	Chen et al. (2018) [49]
Ion exchange	Nitrate	Water	Effectiveness, low operating cost, and simplicity	Dependence on the structure of the resin and the environment of the solution	Kalaruban et al. (2016) [51]
Oxidation	PAHs	Water	Effectiveness, simplicity, and rapidity	Use of chemicals	Antošová et al. (2020) [53]
Photocatalysis + biodegradation	Dyes	Water	Low operating cost and simplicity	Establishment of a favorable environment for the development of bacteria	Waghmode et al. (2019) [55]
Electrochemical treatments	Heavy metals	Water	Effectiveness, low operating cost, and simplicity	Dependence on electrode materials and their high cost	Sharma et al. (2019) [54]

Finally, adsorption is another way to remove pollutants from water. This method is promising, especially since it can be carried out using biomass or agricultural wastes for valorization (bio-based adsorption). It is a non-destructive and environmentally friendly technique that offers high efficiency, simple operation, and easy regeneration. The adsorption capacity depends on the environment of the solution, the pollutants, and the adsorbent [48]. This paper presents a review of the studies that have been conducted on this emerging topic, preceded by a presentation of the parameters involved in this process.

## 5. Factors Affecting Pollutants Adsorption

**Figure 3:**
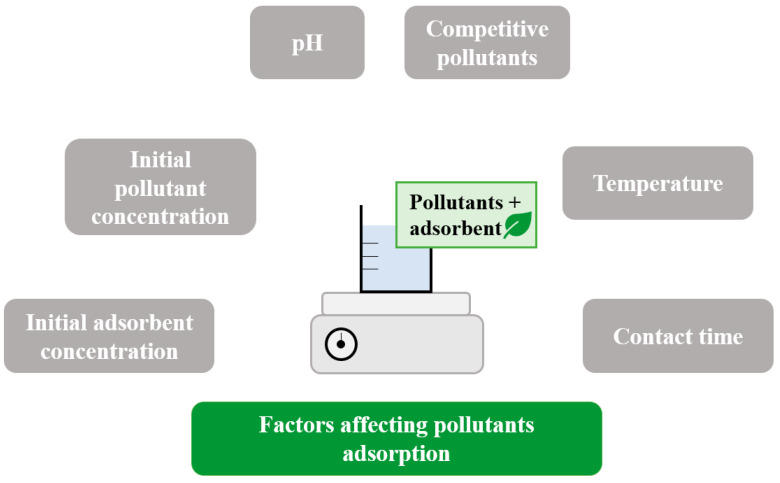
Summary of factors affecting pollutants adsorption on bio-based adsorbent.

The adsorption depends on various parameters that should be studied for each adsorbent–pollutant case. As summarized in Figure 3, the adsorption phenomenon is dependent on pH, temperature, contact time, the initial concentrations of pollutant and adsorbent, and competition with other pollutants for the adsorption sites. 

### 5.1. Effect of Temperature

Generally, increasing the temperature is favorable for adsorption processes. The kinetic energy increases while the viscosity of the solution decreases, leading to a higher diffusion rate and improving the pollutant retention onto the adsorbent [6]. For example, activated carbon from the combination of brown algae and zinc chloride (chemical activating agent) has been synthesized by Nguyen et al. (2022) to remove the synthetic antibiotic ciprofloxacin. The maximum adsorption capacity, calculated by the Langmuir model, increased from 192.3 mg·g^−1^ to 250.0 mg·g^−1^, when the temperature changed from 5 °C to 25 °C, respectively [57]. It was also noted by Zhang et al. (2017), who used the alga *Ulva prolifera* as a potential adsorbent for phenanthrene. This may be due to the direct influence of temperature on the cell chemical composition, nutrient uptake, and thus growth rate of the algae mentioned [58]. Some authors have noted a plateau for the adsorption rate when increasing the temperature. For instance, Biswal et al. (2022) worked with an adsorbent coming from the fruit of a Kendu forest tree (40–90 °C) and noticed a stable value of about 90% of tartrazine dye adsorption at 70 °C [6].

However, in some cases an increase in temperature has no effect or decreases the adsorption capacity. No effect of temperature on adsorption was noted by Foletto et al. (2017), who tested the adsorption of amaranth by iron-based magnetic adsorbent between 25 and 65 °C [59]. In their study, Wang et al. (2020) worked with activated carbon derived from mandarin seed waste to remove six carbamate pesticides from water [60]. Three of the six pesticides (carbaryl, methiocarb, and pirimicarb) had excellent removal rates, between 95% and 100% regardless the temperature, while the rates of the other three (metolcarb, isoprocarb, and bendiocarb) decreased up to 30% when the temperature was increased from 20 °C to 60 °C. The authors supported these results with a thermodynamic study, which revealed an exothermic character of the adsorption process. Similarly, Nizam et al. (2021) worked on the use of powdered activated carbon from rubber seed and its shell to remove dyes from water [61]. They noted a decrease of the adsorption rate when the temperature increases, due to the exothermic character of such adsorption. A reduction in the active surface of the adsorbent due to alteration of the active sites at elevated temperature was one potential justification for these results. 

### 5.2. Effect of pH

The pH is an essential parameter for the removal of pollutants as it can drive the interactions between the adsorbent and the adsorbate. Indeed, it can affect the surface charge, the functional groups of the adsorbent and the pollutants’ charge in solution [7].

There is no specific pH rule, it is pollutant- and adsorbent-dependent. Especially for heavy metals, in some cases the pH improves the removal rate. For example, Es-sahbany et al. (2022) worked on using clay to remove Cu(II), Co(II), Pb(II), and Ni(II), they showed that higher pH was beneficial in terms of adsorption capacity [7]. High pH led to the deprotonation of surface hydroxyls and improved the adsorption capacity thanks to electrostatic interactions between the clay and heavy metals. An et al. (2022) also worked on heavy metal removal and developed a co-system consisting of *Pseudomonas hibiscicola* strain L1 immobilized on peanut shell biochar [16]. They studied the effect of pH on the removal of Cu(II), Cr(IV), Ni(II), and nitrate, and the results differed depending on the pollutant studied. For Cu(II) and Ni(II), the recovery ratio increased when the pH increased (between pH 5 and 8) except at pH 8 where it decreased for Cu(II). They said that at low pH, the repulsive force could restrict the access of metal ions to adsorption sites. Moreover, they stated that depending on the pH, the metal could become solid and thus precipitate. According to the potential-pH diagrams, at pH 8.0 and 5.0, Ni(II) precipitated as Ni(OH)_2_ and Cu(II) as Cu(OH)_2_, respectively. Conversely, for Cr(VI), the ratio decreased from 55% to 30% when the pH increased from 5 to 8. Cr (III) is present as CrOH^2+^ at pH 5 but it switched to Cr(VI) as CrO_4_^2−^ at pH 8, which competed with the hydroxide anion in alkaline condition. Concerning nitrates, the ratio remained stable at a value of about 70%. In addition, the adsorption capacity of *Cornulaca monacantha* stem and biomass-based activated carbon has been examined by Sharma et al. (2018) to remove Congo red dye [62]. Additionally, for this adsorbent, the removal rate decreased with increasing pH. The authors have a similar interpretation to what was reported by An et al. (2022) [16]. As a matter of fact, the deprotonation of the adsorbent surface resulted in a significant negative charge, and in their case, this resulted in a repulsion between the anionic Congo red dye and the adsorbent. Another case with pesticides, Gupta et al. (2011) demonstrated that the adsorption of three pesticides (methoxychlor, methyl parathion, and atrazine) on chemically and thermally modified waste rubber tires decreased with increasing pH [63]. In this case, the deprotonation of the functional groups of the adsorbent (carbonyl and hydroxyl groups) led to strong electrostatic repulsion between the adsorbent and the pollutants, resulting in a decrease in the diffusion and adsorption of pesticides.

All these results show the complexity of the effect of pH on the adsorption of pollutants on bio-based adsorbents.

### 5.3. Effect of Contact Time

The contact time is one of the most important parameters, especially when studying adsorbent efficiency [64]. It defines the average contact time necessary to reach the equilibrium of pollutant concentrations between the aqueous solution and the adsorbent surface. Moreover, it allows to carry out a kinetic study on the pollutant’s adsorption, which will allow to determine the adsorption mechanisms (see section Kinetic models). Typically, the adsorption rate increases rapidly initially due to high site availability before stabilizing. The stabilization is due to a decrease of vacant sites and sometimes to a repulsion between the adsorbed molecules and those still present in solution, in particular for ionic species as dyes [5,61]. It is important to note that a significant difference exists regarding the equilibrium time between the different types of pollutants, especially between heavy metals and PAH. In fact, several tens of hours are necessary for the elimination of PAHs (between 6 h for fluoranthene and 120 h for acenaphthylene), whereas a few hours are required for metals. 

### 5.4. Effect of Initial Pollutant Concentration

The initial pollutant concentration is another important parameter. By varying only the initial pollutant concentration, it is possible to determine the type of adsorption, in particular thanks to different models (see section Isotherm models). The maximum adsorption capacity corresponds to the highest quantity of pollutant that an adsorbent can pick. In general, a direct relationship is noticed between the pollutant’s concentration and the vacant binding sites on the adsorbent surface. A decrease in the removal rate when the initial pollutant concentration increases may be due to an excess number of pollutants relative to the number of available adsorption sites or to the increase of the cohesion forces between the molecules in solution [6,65]. For example, Biswal et al. (2022), who worked on dye removal, explained that a decrease in removal rate was likely related to the decrease in solvent polarity of the solution, which can promote hydrophobic interactions between dye molecules [6].

### 5.5. Effect of Initial Adsorbent Concentration

In general, at a constant pollutant concentration, an increase in adsorbent concentration allows a higher number of available vacant sites and thus an increase in the removal rate and a decrease in the adsorption capacity [61,62,63]. Indeed, as indicated by Raghuvanshi et al. (2004), when only the adsorbent dose is increased, there is a less proportional increase in pollutant adsorption resulting in a lower use of the adsorbent’s adsorption capacity [66]. However, the rate eventually reaches a plateau from one adsorbent dose. Indeed, the number of sites that adsorb the pollutants is greater than the number of pollutants, so an equilibrium is reached. It is therefore necessary to determine the optimal adsorbent concentration in order to avoid an unnecessary amount and waste of it [61].

### 5.6. Effect of Competition with Other Pollutants

The phenomenon of pollutant adsorption on biomass is moderately complex, since only the parameters mentioned above and the adsorbent–adsorbent interactions are involved. When the pollutant solution contains more than one pollutant, then a competition effect and a selectivity order occur. Few studies have investigated this effect; the authors who have studied it have generally shown that a competition exists whatever the type of adsorbent and pollutants [67,68,69]. For example, Ali et al. (2018) worked on the use of a modified algal biomass for the removal of a mixture of five pharmaceuticals [68]. The adsorption capacity of the analgesic named tramadol has decreased from 47 mg/g to 42 mg/g when four other drugs were added. Most notably, according to Kajeiou et al. (2020), the maximum adsorption capacities of three metals on flax fibers decreased when the solutions contained one, two, and three metals at the same initial concentration [69]. In effect, the maximum adsorption capacities decreased from 23.2, 7.8, and 4.6 mg/g, for Pb(II), Cu(II), and Zn(II) in a single metal ion solution to 17.0, 3.6, and 0.5 in a ternary solution, respectively. Similar results have been reported by Selim et al. (2019) [67] for Cu(II) and Zn(II) and by Sellaoui et al. (2019) [70] for Hg(II), Pb(II), and Zn(II). An antagonistic adsorption of Hg(II), Pb(II), and Zn(II) was noticed when switching from solutions containing only one metal to those containing all three. In particular, Zn(II) was the metal most affected by the competition effect as its adsorption capacity collapsed by 66%. This finding led to a change in the order of metal selectivity between the two types of solution. For mono-metallic solutions, Hg(II) is the most adsorbed metal while Pb(II) is the least, while for multi-metallic solutions, Zn(II) is the least adsorbed [70]. A decrease in the adsorption capacity of modified sugarcane bagasse biochar was also demonstrated for nitrate in the presence of other ions (such as phosphate, carbonate, sulfate, and chloride) [8]. These results revealed the existence of competition of pollutants for adsorption sites of different biomasses.

## 6. Kinetic and Isotherm Models

Isotherm and kinetic models appeared important to understand the mechanisms of pollutant adsorption on biomass [10]. Febrianto et al. (2008) wrote a review on this subject, where the numerous existing models were listed [71]. This section provides a quick summary of the different models often used during pollutant adsorption studies.

### 6.1. Kinetic Models

#### 6.1.1. Adsorption Models

Adsorption kinetics can be used to possibly understand the step that determines the adsorption rate [72]. The study is performed at different equilibrium contact times and at a constant temperature. The kinetic study provides an understanding of the adsorption mechanism and rate control steps [71]. The most common models will be presented: pseudo-first-order (PFO), pseudo-second-order (PSO), Elovich, and Weber–Morris models.

The pseudo-first-order, also named the Lagergren first-order model can be found as a derivative, but more commonly in the following integrated Equation (1):

(1) $$q_{t}=q_{e}\left(1-\exp\left(-k_{1}t\right)\right)$$

and the linear form (Equation (2):

(2) $$\ln\left(q_{e}-q_{t}\right)=\ln q_{e}-k_{1}t$$

where q_t_ is the adsorption capacity (mg/g) after the contact time t, q_e_ is the equilibrium adsorption capacity (mg/g), and k_1_ is the first-order adsorption rate constant.

If the experimental results fit well with this model, it implies a physisorption governed by diffusion steps between the pollutants and the adsorption sites of the biomass [73]. 

As for the pseudo-first order, the initial pseudo-second order equation is described by a derivative and more commonly in the following integrated form (3):

(3) $$\frac{1}{q_{e}-q_{t}}=\frac{1}{q_{e}}+k_{2}t$$

and the linear form (4):

(4) $$\frac{t}{q_{t}}=\frac{t}{q_{e}}+\frac{1}{k_{2}q_{e}^{2}}$$

where q_t_ is the adsorption capacity (mg/g) after the contact time t, q_e_ is the equilibrium adsorption capacity (mg/g), and k_2_ is the second-order adsorption rate constant.

If the experimental results are consistent with this model, it implies chemisorption and the adsorption rate of the pollutants is then linearly related to the square of the vacant adsorption sites on the adsorbent surface [73]. 

The Elovich model is also initially described by a derivative equation, but it has been simplified by Chien and Clayton [74] (5):

(5) $$q_{t}=\frac{\ln\alpha \beta }{\beta }+\frac{1}{\beta }\mathrm{lnt}$$

where q_t_ is the adsorption capacity (mg/g) after the contact time t, α is the initial concentration rate (mg/g/min), β is the adsorption constant (g/mg), and t is the time.

If the experimental results fit well with this model, then chemisorption is involved and the adsorption rate of the pollutant decreases exponentially as the amount of adsorbed pollutant increases.

The Weber–Morris kinetic model was initially described by Weber and Morris in 1962 [75], in the following form (6):

(6) $$q_{t}={K\times t}^{1/2}+C$$

where q_t_ is the adsorption capacity (mg/g) after the contact time t, K is the intraparticle diffusion constant (mg·g^−1^ min^−1/2^), and C (mg·g^−1^) is a constant related with diffusion resistance.

#### 6.1.2. Desorption Model

Few studies have been conducted on desorption kinetics. A recent study by Kajeiou et al. (2021), based on the study by Njikam and Shiewer (2012), proposes to characterize the desorption of pollutants by the following models modified from the classical pseudo first- and second-order models [76,77] (7):

- Pseudo-first-order
  (7) $$q_{t}={q_{\mathrm{Rf}}+(q}_{e\;}-q_{\mathrm{Rf}})\;e^{-k_{1,\mathrm{ds}}t}$$
  where q_t_ is the adsorption capacity (mg/g) after the contact time t, q_Rf_ is an additional parameter considering the quantity of final retained pollutant onto adsorbent at the end of the desorption process, q_e_ (mg/g) is the amount adsorbed per mass of adsorbent at equilibrium, and $k_{1,ds}$ is the first-order desorption rate constant (min^−1^) (8).
  (8) $${\;q}_{t}=q_{\mathrm{Rf}}+\frac{q_{e}-q_{\mathrm{Rf}}}{1+(q_{e}-q_{\mathrm{Rf}})\;k_{2,\mathrm{ds}}t}$$

- Pseudo-second-order
  where q_t_ is the adsorption capacity (mg/g) after the contact time t, q_Rf_ is an additional parameter considering the quantity of final retained pollutant onto adsorbent at the end of the desorption process, q_e_ (mg/g) is the amount adsorbed per mass of adsorbent at equilibrium, and $k_{2,ds}$ is the second-order desorption rate constant (min^−1^).

### 6.2. Isotherm Models

Adsorption isotherms allow to understand the distribution of the pollutant between the polluted liquid phase and the solid phase of the biomass. The study is performed at different equilibrium concentrations and at a constant temperature. They allow to give a relation between the quantity adsorbed by a weight adsorbent unit at the equilibrium [8]. 

#### 6.2.1. Single Pollutant

The most common models will be presented: Freundlich, Langmuir, and Brunauer–Emmett–Teller (BET) models. They are used to describe the behavior of a single pollutant at once.

Herbert Freundlich hypothesized that the surface of the adsorbent may be heterogeneous. Furthermore, the extent of adsorption would vary linearly with pressure at small intervals. He thus proposed this empiric Equation (9) to express the model [78]:

(9) $${q_{e}=K_{f}C_{e}}^{\frac{1}{n}}$$

The resulting linear form is Equation (10):

(10) $$\log q_{e}=\log K_{f}+\frac{1}{n}\log C_{e}$$

where q_e_ (mg/g) is the amount adsorbed per mass of adsorbent at equilibrium, K_f_ (mg/g)(L/mg)^1/n^ and n are Freundlich constants, and C_e_ (mg/L) is the concentration of pollutant at equilibrium.

This model is one of the most-used isotherms to describe the adsorption equilibrium. It implies that the energy present at the adsorbent surface is heterogeneous and that each adsorption site could contain several molecules in thickness, which would allow reversible adsorption [79]. The adsorption sites with the highest energy would be occupied first. However, the adsorption equilibrium data are not predictable by the model for extreme, low or high concentrations [71].

In contrast, the Langmuir model is based on homogeneous energy and energetically equivalent sites on the adsorbent surface. Therefore, each adsorption site can contain only one molecule in thickness and the pollutant concentrates only on the unoccupied sites [71]. The non-linearized equation is the following Equation (11):

(11) $$q_{e}=\frac{q_{\max}K_{L}C_{e}}{1+K_{L}C_{e}}$$

where q_e_ (mg/g) is the amount of pollutant adsorbed per mass of adsorbent at equilibrium, q_max_ (mg/g) is the maximum adsorption capacity, K_L_ (L/mg) is the adsorbent/adsorbate interaction constant, and C_e_ (mg/L) is the concentration of pollutant at equilibrium.

The resulting linear form is Equation (12):

(12) $$\frac{C_{e}}{q_{e}}=\frac{1}{q_{\max}}C_{e}+\frac{1}{K_{L}q_{\max}}$$

In addition, some adsorption behaviors do not fit these two classical models, so various models originally developed for the study of gas adsorption have been introduced in an attempt to correlate the adsorption process of pollutants. To accomplish this goal, the equations may contain more than one fitting parameter. Such is the case of the Brunauer–Emmer–Teller model. In contrast to the Langmuir model, it assumes that the first layer of adsorbed pollutants can be considered as another layer available to host a new adsorption phase. In that case, the isotherm is able to keep increasing instead of stabilizing at a certain saturation value, as the Langmuir isotherm would have done [71]. The simplified equation is the following (13):

(13) $$q_{e}=q_{\max}\frac{BC_{e}}{\left(C_{e}-C_{s}^{*}\right)\left[1+(B-1)\left(\frac{C_{e}}{C_{s}^{*}}\right)\right]}$$

where q_max_ (mg/g) is the maximum adsorption capacity, B is a BET constant, and C_e_ and C_s_^*^ are the pollutant concentration in the solution at equilibrium and saturation, respectively (mg/L).

#### 6.2.2. Multiple Pollutant

Recently, the models classically used to describe the adsorption of a single pollutant have been modified to describe the adsorption behavior of several pollutants simultaneously, mainly bi-element solutions. The classical Langmuir model is the main one modified (non-modified Langmuir, modified Langmuir, competitive Langmuir, uncompetitive Langmuir, and partial competitive Langmuir isotherms).

Non-modified Langmuir is a model developed on the adsorption data obtained with the classical Langmuir model for a single pollutant [80]. The non-linearized equation is the following Equation (14):

(14) $$q_{e,i}=\frac{q_{\max,i}K_{L,i}C_{e,i}}{1+\sum_{j=1}^{2}K_{L,j}C_{e,j}}$$

where q_e_ (mg/g) is the amount of pollutant adsorbed per mass of adsorbent at equilibrium for single pollutant, q_max_ (mg/g) is the maximum adsorption capacity for single pollutant, K_L_ (L/mg) is the adsorbent/adsorbate interaction constant for single pollutant, and C_e_ (mg/L) is the concentration of single pollutant at equilibrium. For the competitive Langmuir isotherm, Equation (14) is also employed; however, the parameters used are those of multiple pollutant solutions.

The modified Langmuir model is expanded from the previous model with a new parameter *η* that is estimated from multiple data, and is expressed by Equation (15) [80]:

(15) $$q_{e,i}=\frac{q_{\max,i}K_{L,i}{(C}_{e,i}/\eta_{i})}{1+\sum_{j=1}^{2}K_{L,j}(C_{e,j}/\eta_{j})\;}$$

where q_e_ (mg/g) is the amount of pollutant adsorbed per mass of adsorbent at equilibrium for single pollutant, q_max_ (mg/g) is the maximum adsorption capacity for a single pollutant, K_L_ (L/mg) is the adsorbent/adsorbate interaction constant for a single pollutant, C_e_ (mg/L) is the concentration of a single pollutant at equilibrium, and η is the estimated parameter from multiple data.

The uncompetitive Langmuir model was developed for binary solutions. This model assumes that both pollutants can be attached to a single adsorption site simultaneously. The equation is the following (16) [81]:

(16) $$q_{e,i}=q_{\max}\left(\frac{K_{L,i}C_{e,i}+K_{L,\mathrm{ij}}C_{e,i}C_{e,j}}{1+K_{L,i}C_{e,i}+K_{L,j}C_{e,j}+K_{L,\mathrm{ij}}C_{e,i}C_{e,j}}\right)$$

where q_e_ (mg/g) is the amount of pollutant adsorbed per mass of adsorbent at equilibrium for pollutant in multiple solution, q_max_ (mg/g) is the maximum adsorption capacity for pollutant in multiple solution, K_L_ (L/mg) is the adsorbent/adsorbate interaction constant for pollutant in multiple solution, and C_e_ (mg/L) is the concentration of pollutant in multiple solution at equilibrium.

Partial competitive Langmuir model was also developed for binary solutions. This model assumes that a pollutant may be fixed to an unoccupied adsorption site or to a site already occupied by another pollutant [81]. The equation is the following (17):

(17) $$q_{e,i}=q_{\max}\left(\frac{K_{L,i}C_{e,i}+K_{L,\mathrm{ij}}C_{e,i}C_{e,j}}{1+K_{L,i}C_{e,i}+K_{L,j}C_{e,j}+{(K_{L,i}K}_{L,\mathrm{ij}}{+{K_{L,j}K}_{L,\mathrm{ij}})\;}_{}{C_{e,i}C}_{e,j}}\right)$$

where q_e_ (mg/g) is the amount of pollutant adsorbed per mass of adsorbent at equilibrium for pollutant in multiple solution, q_max_ (mg/g) is the maximum adsorption capacity for pollutant in multiple solution, K_L_ (L/mg) is the adsorbent/adsorbate interaction constant for pollutant in multiple solution, and C_e_ (mg/L) is the concentration of pollutant in multiple solution at equilibrium.

Finally, the Freundlich model was also modified to characterize the behavior of pollutant in solution simultaneously [82]. The equation is the following (18):

(18) $$q_{e,i}=\frac{K_{f}C_{e,i}^{1/n_{i}+x_{i}}}{C_{e,i}^{x_{i}}+y_{i}C_{e,j}^{z_{i}}}$$

where q_e_ (mg/g) is the amount adsorbed per mass of adsorbent at equilibrium, K_f_ (mg/g)(L/mg)^1/n^ and n are Freundlich constants belong to the single pollutant Freundlich model, C_e_ (mg/L) is the concentration of pollutant at equilibrium, and x, y, and z are parameters determined from multiple pollutant data.

## 7. Adsorption Phenomena Involved in the Retention of Pollutants

**Figure 4:**
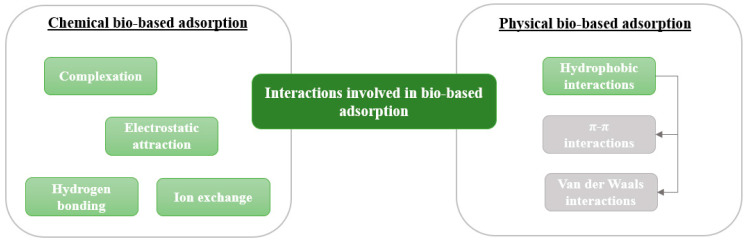
Summary of interactions involved in bio-based adsorption of pollutants. Modified from Ref. [89].

During the adsorption of different classes of pollutant, chemical and physical interactions are involved, as listed in Table 2 and Figure 4. In the cited studies, the explanations of the interactions implied in the adsorption phenomenon are generally assumptions of the authors.

Concerning chemical adsorption, pollutants can react with the adsorbent or have chemical complexation effects [88]. Different types of interactions can be mentioned: electrostatic attraction, ion exchange, complexation, or hydrogen bonding [7]. In the case of electrostatic attraction, if the pollutants and the adsorbent have opposite charges, they spontaneously attract each other. In the case of ion exchange, an ionic bond is established with the exchangeable ions present on the adsorbent surface. Pollutants in ionic or ionizable form are therefore probably adsorbed via these processes. Regarding hydrogen bond, it is an intermolecular or intramolecular force involving a hydrogen atom and an electronegative atom such as oxygen or nitrogen. In these three cases, a reversible adsorption occurs. According to various studies cited in Table 2, these processes or their combination with physical processes can explain the retention of dyes, heavy metals, nitrates, pesticides, and pharmaceuticals by different types of bio-sourced adsorbents. 

Among physical processes, hydrophobic interaction, including π-π and van der Waals interactions, can be cited to understand pollutant adsorption [10]. The π-π interactions engage the lobes of an atomic orbital of the two different molecules while the van der Waals interactions employ weak electrical interactions between two atoms or molecules; they are called dipoles. Due to the benzene rings of PAHs, their adsorption involves this type of interaction, while for heavy metals and nitrates, which are in ionic form, chemical interactions are required. This was particularly studied by Akinpelu et al. (2021), who worked on the use of biomass derived from dead leaves of Halodule uninervis to remove PAHs from contaminated water [10]. Only hydrophobic interactions have been mentioned by the authors to justify the adsorption of pollutants. They added that the intensity of these interactions increased with the number of rings of the PAHs (i.e., their hydrophobicity).

## 8. Bio-Based Adsorption to Remove Pollutants from Water

As previously mentioned, the adsorption of pollutants via biomass is a promising avenue under development. Regarding the biomass used, different types have been reported: agricultural waste, microbial biomass, algae, rock, and mineral materials, or activated carbon/biochar. Therefore, this section presents a review of the biomass type employed for pollutant removal, but it also includes a summary of parameters affecting adsorption in Table 3 It is classified by biomass used and contains the kinetic and isotherm models used to describe the adsorption of the different pollutants present in water.

### 8.1. Agricultural Waste

Unmodified and modified biomasses from agricultural waste are frequently employed to clean up water contaminated by various pollutants. Unmodified wastes were tested as potential adsorbents in numerous studies [6,10,90,93]. For example, Cu(II) was removed at 9.51 mg/g by carnauba straw and at 1.73 mg/g by cashew leaf with an equilibrium time of 120 min by Pereira and colleagues (2021). Abbar et al. (2017) demonstrated that flax fiber tows showed an adsorption capacity of 9.9 mg/g for Cu(II), 10.7 mg/g for Pb(II), and 8.4 for Zn(II), within one hour [92]. This study was extended by Kajeiou et al. (2020), who demonstrated that a competition effect existed for these three metals at the adsorption sites of the fibers [69]. Furthermore, acenaphthylene, phenanthrene, and fluoranthene were recovered at lower capacities near 2 mg/g by seagrass leaf powder in the study of Akinpelu et al. (2021). The authors of this study, and others, noted that PAH removal efficiency increased as the number of rings increased [10,72].

In addition, some authors have tried to chemically modify natural agricultural wastes to improve their adsorption capacity. Biswal et al. (2022) tested a natural powder obtained from the fruit coat of a Kendu tree and a cellulosic nano-adsorbent modified from it for tartrazine dye removal. The modified adsorbent removed more than 95%, while the natural powder removed about 75% [6]. Furthermore, acid treatment (using citric, hydrochloric, nitric, or sulfuric acids) has also often been used in an attempt to increase the removal capacity of several biomasses, such as coconut shell, *Moringa oleifera* seed husk, peat, and sawdust [11,83,86]. For instance, Xi et al. (2014) used acid hydrolysis to modify various natural materials, such as bamboo wood, with the aim of removing the polar portion of the surface (i.e., polysaccharides) and improving PAHs removal [90]. The removal of polysaccharides increased the sorption capacity of plant residues; however, pollutant removal was found to be faster for raw adsorbents. Another type of modification was tested by Abdelhameed et al. (2018), they used the synthetic polymer polyethyleneimine to increase the active sites of natural cotton and wood-based fabrics [87]. Initially, untreated wool fabrics showed higher adsorption amounts than cotton and better affinity for pirimiphos-methyl than for monocrotophos. Finally, the structural modification further improved the recovery of both pesticides. Indeed, the maximum adsorption capacity of wood was increased from 200.0 mg/g to 625.0 mg/g for pirimiphos-methyl and from 153.9 to 500.0 mg/g for monocrotophos. Thus, this modified adsorbent has the best adsorption capacity for pesticides among those listed in Table 3. A good capacity was also found for biochar derived from used rubber tires, but remains about five to six times lower, with values close to 100 mg/g [63]. In general, pesticides of different families considered in these studies predominantly followed the pseudo-second order, reflecting chemisorption, and the Langmuir model, reflecting homogeneous adsorption sites.

### 8.2. Microbial Biomass

In recent years, new studies have focused on the adsorption capacity of microbial biomass. For example, Vasiliadou et al. (2016) tested the single and combined ability of two white rot fungi (*Trametes versicolor* and *Ganoderma lucidum*) to remove 13 pharmaceutical contaminants from water [94]. Five of the thirteen pollutants were fully removed from waters regardless of the fungi combination. For the other eight, the rates were better with the combination but remained between 15% and 41%. They stated that pollutant removal was possible through both extracellular and intracellular oxidation mechanisms, which was a change from the two types of adsorption seen since the beginning of this review. In addition, they tried to produce potential feedstock for biodiesel production via the valorization of fungal sludge generated during the elimination process. They were able to convert 30% of the initial dry mass of the strains, however, further studies need to be conducted to determine the effectiveness. Sharma et al. (2018) encapsulated iron oxide nanoparticles and a strain of *Agrobacterium fabrum*, in calcium alginate to make a nano-adsorbent for methylene blue dye removal. Using this innovative adsorbent, they were able to achieve an adsorption capacity of 91 mg/g in one hour. In addition, they studied the possibility of reusing it, and they showed that after four consecutive cycles of adsorption and desorption, the efficiency was still 85% [18].

### 8.3. Algae

Living organisms, such as algae, also have interesting properties for the adsorption of pollutants. As for other biomasses, both modified and unmodified were tested. For the unmodified, Zhang et al. (2017) determined that the alga *Ulva prolifera*, involved in the green tide phenomenon, had the ability to remove phenanthrene from waters [58]. In fact, they demonstrated that the use of this alga allowed to decrease the phenanthrene concentration from 10.0 µg L^−1^ to 0.80 µg L^−1^ during the 31-day incubation process. The presence of proteins, polysaccharides, or lipids on the surface of their cell walls seemed to be involved in the adsorption of pollutants. However, they showed that 50% of the phenanthrene loss is due to abiotic loss characterized by photodegradation and volatilization. Additionally, they tried to perform the same experiments by imposing a heat treatment to kill the algae. The results were less conclusive as the phenanthrene concentration decreased from 10.0 µg/L to 2.71 µg/L.

Chemical modification of this type of biomass is also considered. For example, an alkaline solution was used by Ali et al. (2018) to modify the alga *Scenedesmus obliquus* to adsorb pharmaceutical compounds from waters [68]. The chemical modification significantly increased the recovery of the analgesic tramadol from 20% to 95%, ultimately resulting in an adsorption capacity of 140 mg/g. Pyrolysis can also increase the adsorption capacity of a biomass (see section Biochar and activated carbon). An adsorption capacity of 250 mg/g was found by Nguyen et al. (2022) to remove another pharmaceutical compound, ciprofloxacin, by a biochar derived from brown algae [57]. Additionally, the study of Ali et al. (2018) once again demonstrated the competitiveness of pollutants for adsorbent active sites [68]. They studied this aspect with a mixture of five pharmaceutical compounds (cefadroxil, paracetamol, ciprofloxacin, tramadol, and ibuprofen). Within this mixture, tramadol experienced a competitive effect as the equilibrium absorption capacity was about 40 mg/g compared to the others ranged between 39 and 68 mg/g [68].

### 8.4. Rock and Mineral Materials

Rock and mineral materials were evaluated as another type of natural biomass for water treatment because they may be naturally microporous, such as zeolite, and therefore prone to adsorption. Calcite, zeolite, sand, clay, and synthetic cancrinite have been tested as potential adsorbents for heavy metal and nitrate removal [7,13,67]. Indeed, Reddy et al. (2014) compared the adsorption capacity of four filter materials for the recovery of nitrate, phosphorus, and six heavy metals. Their results showed that the filters studied had different removal efficiencies depending on the contaminant and the coexistence of other contaminants. For most filter materials, the efficiency decreased when several contaminants were present in solution. For example, the adsorption efficiency of Zn(II) by calcite decreased when it was in mixed solution. Selim et al. (2019) concluded the same on their work on the use of cancrinite synthesized from crude muscovite to remove Cu(II) and Zn(II) from contaminated water [67]. This remark is not effective in all cases. Concerning the four filter materials, phosphorus, Cd(II), Cu(II), Pb(II), and Cr(VI) were recovered at higher efficiencies when they were in mixed solutions, especially Cu(II), which increased its efficiency from about 30% to 70% [13]. Wang et al. (2019) developed modified calcium montmorillonite clays to remove PCBs from water. They concluded that this adsorbent had potential to reduce exposure to environmental pollutants in food and drinking water [24]. Moreover, the heavy metal adsorption process followed the three kinetic models cited in the kinetic and isotherm models section, i.e., the pseudo-first-order, pseudo-second-order and Elovich models, and the Langmuir and Freundlich models for isotherms.

### 8.5. Biochar and Activated Carbon

The pyrolysis method is another way to modify an organic material. It can be used to increase the matrix porosity of biomass by thermochemical modification, in order to obtain a better removal efficiency of pollutants. Generally, pyrolysis is performed in an oxygen-limited atmosphere, especially under a nitrogen flow [95,96]. Biochar is produced during a step called carbonization in which the biomass is heated to a maximum temperature of about 600 °C. In addition, a chemical or physical activation step is necessary to produce activated carbon. The chemical activation is performed before or after the carbonization step, using chemicals (iron chloride, potassium hydroxide, phosphoric and sulfuric acids, or zinc chloride) [97,98]. On the contrary, physical activation is only realized after the carbonization. The biomass is heated to higher temperature, under a flow of activating agent, such as steam or carbon dioxide. Different parameters impact the quality of the activated carbon generated: the temperature gradient, the final temperature, and the carbonization time, which is the time for which the biomass is heated to this temperature. A summary of the optimal parameters selected from different papers and for various biomass types are grouped in Table 4.

Generally, pyrolysis is performed at temperatures between 300 °C and 900 °C with a gradient of 10 °C/min, and carbonization is carried out between 2 and 4 h. Bio-sourced activated carbon is a promising approach for water treatment, which explains the numerous studies that have been conducted on this subject. This process has been used by many authors to increase the adsorption capacity of agricultural wastes, such as corn straw, rubber seed and shell, sugarcane bagasse, tangerine seed, wheat straw, wood waste, or other wastes [8,60,61,70,85,99]. As shown in Table 4, flamboyant biomass biochar was prepared by Sellaoui et al. (2019) through pyrolysis at 600 °C for 2 h under nitrogen atmosphere. Similarly, Wang et al. (2020) produced biochar from tangerine seeds to remove pesticides at the same carbonization temperature as the above study but with a carbonization time of 4 h. In their study, they demonstrated that carbonization time has a significant influence on biochar removal performance, by searching for optimal pyrolysis conditions, and that the adsorption capacity of biochar was higher than that of the raw material [60]. This finding was extended by Suo et al. (2019), who found that the adsorption rate of pesticides increased with the activation step from less than 60% to more than 95% by activating corn starch with phosphoric acid before pyrolysis [85]. Thus, as Li et al. (2014) reported, chemical activation may generate a larger number of active sites on the adsorbent surface due to a higher surface area and pore volume [99].

Pyrolysis can be performed on any type of raw material. For example, as shown in Table 4, Nguyen et al. (2022) carbonized brown algae and studied the gases emitted during carbonization using Py-GC/MS. They showed that many compounds such as benzene or toluene were released. They concluded that pyrolysis could also allow the recovery of valuable gaseous products [57]. This method is also a potential way of revalorizing old products from transformed natural biomass such as used tires. As a matter of fact, Gupta et al. (2011) converted waste rubber tires into activated carbon by activation with potassium hydroxide followed by carbonization at 900 °C for 2 h. Finally, activated carbon is able to remove the pesticides methoxychlor, atrazine, and methyl parathion in 1 h with adsorption capacities of 112.0 mg/g, 104.9 mg/g, and 88.9 mg/g, respectively. Regarding these results, they noted a direct correlation between the adsorption capacity and the octanol–water partition coefficient (Log Kow). Notably, methyl-parathion is the least hydrophobic pesticide as it has the lowest Log Kow value; this translates into limited adsorption [63]. In addition, Guo et al. (2016) worked on the use of coconut shell-based activated carbon to remove dioxins with a maximum adsorption capacity of 600 mg/g [91].

A combination of biochar and another material is an additional approach investigated to obtain an effective adsorbent. Bamboo–biochar composite with montmorillonite has been developed by Viglašová et al. (2018). The biochar was obtained by applying the pyrolysis parameters shown in Table 4, and the composite was made by immersing it in a clay suspension. At pH 4, the addition of montmorillonite increased the adsorption capacity from 5 mg/g for the biochar to 9 mg/g for the composite for nitrate removal. Modified biochar has often been used as a potential adsorbent for nitrate. The adsorption capacity cited was lower than that of biochar derived from sugarcane bagasse found by Divband Hafshejani et al. (2016), which was 28 mg/g [8]. 

As another example, in the study by An et al. (2022) a co-system of *Pseudomonas hibiscicola* strain L1 immobilized on peanut shell biochar was created to remove heavy metals, including Ni(II). The combination with biochar significantly increased Ni(II) recovery from 15.5% with the L1 strain adsorbent to 81.2%. They observed a complexation of Ni(II) on the surface of the co-system by the functional groups, which explains its removal from wastewater. 

As shown in Table 3, activated carbon or biochar was the type of adsorbent that allowed the greatest amount of dye and nitrate removal. The activated carbon produced by Nizam et al. (2021) had a maximum adsorption capacity of 769.23 mg/g and 458.43 mg/g for methylene blue and Congo red, respectively [61]. In other studies, the adsorption capacity ranged from 45 mg/g for the iron-based adsorbent from Litchi bark biomass to 91 mg/g for *Agrobacterium fabrum* biomass [18,59]. According to the studies presented in Table 3, the adsorption of dyes followed the pseudo-second-order model, reflecting chemisorption, and the Langmuir and Freundlich models.

## 9. Future Outlooks

Recently, a new planetary limit has been overpassed and concerned chemical pollution [100]. Among the elementary elements of earth, water is especially polluted by compounds coming from an anthropogenic source. Researchers have been trying for years to develop effective and inexpensive means of depollution in order to limit water pollution by micropollutants. For this purpose, the use of bio-based adsorbents, which have many advantages, has been widely explored recently. Some of them had limited adsorption capacities; an option is to develop chemical or physical modification to improve their efficiency, but it increases the environmental impact. Some studies were conducted to determine if the use of microwaves during adsorbent synthesis could increase the specific surface area. The results about synthesis conditions are gathered in a recent review from Ewis and Hameed (2021) focused on naturally occurring adsorbents [101]. Notably, Yagmur et al. (2017) compared the specific surface areas of a commercial activated carbon and tea waste derivatives prepared using microwaves. Their study showed that microwave assistance resulted in a higher specific surface area of the synthesized carbon than the commercial carbon [102]. Ibrahim et al. (2021) wrote a review on another type of adsorbent, metal-organic frameworks, which can be used for soil, air, and water remediation. Those innovative carbonaceous nanomaterials offer large specific surface areas with surfaces covered with oxides, allowing good adsorption [103]. Researchers are also increasingly interested in polysaccharide-based magnetic adsorbents, as presented in the review of Wang and You (2021). In situ and ex situ synthesis processes have been developed to synthesize this type of composite. This method has many advantages such as possible regeneration, low environmental impact, and good cost effectiveness of the adsorbent [104]. These innovative adsorbents are susceptible to be effective for water depollution, and continuous studies must be carried out, notably on real river discharges, in order to determine their effectiveness in real conditions.

Some limiting effects of the use of biomass for depollution must be highlighted. For example, during the pyrolysis process of a biomass, many toxic volatile compounds are released, and their treatment can be a problem. The impact of this treatment should not outweigh the beneficial effects of biomass recovery. Michal (1976) demonstrated decades ago that pyrolysis of poly(vinyl chloride) could produce hazardous degradation products, including non-negligible amounts of hydrogen chloride and negligible amounts of chloromethane [105]. This study was extended to polyamides; again it was proven that dangerous compounds such as nitriles were released during heating [106]. Although these studies were not conducted on biomass, it is important to understand the issue of degradation products, and studies seem necessary on this subject.

Treatment of polluted adsorbents is another aspect to consider. Treatment of polluted activated carbons has already been studied using heat and biological regeneration. A recent study was devoted to the biological regeneration of activated carbons and indicates that the use of micro-organisms to regenerate activated carbon allows, in particular, to avoid logistic difficulties. This process is safer, less expensive, and more environmentally friendly than other conventional regeneration processes such as steam regeneration and chemical regeneration [107]. Concerning the other polluted adsorbents, it is important that researchers consider their future in the upcoming studies on this topic. This aspect may be important from an ecological point of view as well. As a matter of fact, it is essential to understand that a desorption of the pollutants may be generated, in particular if the physicochemical conditions change during the adsorption process. This can lead to a real risk of releasing previously adsorbed pollutants into the natural environment. For example, Kajeiou et al. (2021) investigated the desorption of three heavy metals (Cu(II), Pb(II), and Zn(II)) with acidic, basic, and neutral agents previously adsorbed on flax fibers. They were able to desorb significant percentages of the metals in the presence of acids or in a metal solution. Indeed, between 73% of Pb(II) and 100% of Zn(II) attached to flax fibers using nitric and hydrochloric acids were desorbed. In addition, negligible percentages were released in the presence of ultrapure water (between 0.7% and 7%) [77]. Moreover, Yadav et al. (2021) wrote a review on biomass-based composites, and they included a section on regeneration. They cited numerous studies using acids, bases, and other chemicals to successfully recover different types of pollutants, such as heavy metals and dyes [108]. For example, Altun and Ecevit (2020) used biochar derived from cherry kernel shell mixed with chitosan and iron oxide nanoparticles for Cr(VI) removal. They successfully desorbed 85% of the metal from the biochar and 94% of the composite with sodium hydroxide before investigating the potential reuse of the regenerated biochar [109]. These studies demonstrate how a change in water pH can lead to desorption of metals bound to the biomass and thus to unwanted hazardous release. Studies should be extended to investigate the dangers of releasing pollutants by varying others physicochemical parameters (temperature, ionic strength, etc.). In addition, it may be possible to recover the pollutants adsorbed on the biomass for use in another sector, therefore studies on the quality of the recovered pollutants must be conducted. 

Finally, the development of standards for acceptable concentrations in different types of water is also necessary. These exist for some heavy metals, pesticides, and PAHs, but are not available for dyes and pharmaceuticals. Addressing the maximum allowable concentrations in water is a major challenge to limit adverse effects on human health and the environment.

## 10. Conclusions

Intense human activities have resulted in the pollution of the environment by different types of micropollutants, such as inorganic pollutants, organic pollutants, microbial contaminants, and radioactive and thermal pollutants. Water has become a scarce resource due to climate change and today; the quality of this resource is in danger. Every available tool must be used to protect it. Several methods have been developed to try to limit water pollution, among which adsorption seems promising, due to its high efficiency, availability, and low cost. This article provides a review on the use of adsorbents of various origins for the removal of different types of pollutants: dyes, heavy metals, nitrates, pesticides, pharmaceuticals, and polycyclic aromatic hydrocarbons. Recent works have shown that raw or modified adsorbents are effective for adsorbing part or all of these pollutants at concentrations in the mg L^−1^ range. However, different parameters may influence the quality of adsorption and desorption, such as pH, temperature, presence of other pollutants, etc. Some of these parameters are used to describe mathematical models, which allow to understand the adsorption behavior of pollutants on adsorbents. Classical Langmuir, Freundlich, and BET models were presented, as well as versions developed to understand the adsorption behavior of pollutants when they are multiple in solution. In general, dyes, PAHs, pesticides, and pharmaceuticals considered in these studies predominantly followed the pseudo-second order, reflecting chemisorption. The heavy metal adsorption process followed the three kinetic models cited in the kinetic and isotherm model section. Langmuir and Freundlich isotherms are the most represented for the pollutants adsorbed on the different types of adsorbent. However, few studies are dealing with desorption conditions and the possibility to reuse the adsorbent after several cycles of adsorption/desorption. Although many studies have been conducted on sustainable ways to clean up the environment, we need to successfully implement a circular economy in the major industries that release pollutants in order to limit the production of waste by creating a closed loop system.

## Figures and Tables

**Table 2:** Chemical and physical interactions involved in the adsorption process of pollutants.

	Dyes	Heavy Metals	Nitrates	PAHs	Pesticides	Pharmaceuticals
Electrostatic attraction	x [6,83]	x [7]	x [84]		x [85]	x [11,57]
Ion exchange		x [7,13,16,86]	x [16,84]			
Complexation		x [16]	x [16]			
H-bonding	x [6,61,83]				x [85,87]	x [57]
π-π interaction	x [6,61,83]			x [10]	x [87]	x [57]
Van der Waals interaction				x [10]	x [60,85,87]	

**Table 3:** Biomass and physicochemical parameters used for the removal of pollutants from water (A: algae, AW: agricultural waste, B: biochar and activated carbon, MB: microbial biomass, and R: rock and mineral materials).

Adsorbent	Type of Biomass	Pollutant	Equilibrium Time	Maximum Adsorption Capacity (mg/g)	pH	Temperature (°C)	Kinetic	Isotherm	Important Remarks	Reference
Acid-factionalized Coconut shell	AW	Methylene blue	60 min	50.6	8	-	PSO	Freundlich	By increasing the pH, the adsorption capacity increased.	Jawad et al. (2020) [76]
*Agrobacterium* fabrum biomass	MB	Methylene blue	60 min	91	11	25	PSO	Freundlich	The pH was the most influential parameter and the removal rate decreased with increasing pH. Conversely, an increase in adsorption capacity with increasing initial dye concentration was noted.	Sharma et al. (2018) [18]
Iron-based adsorbent from Litchi peel biomass	B	Amaranth	180 min	44.9	6.2	No effect between 25–65 °C	PSO	BET isotherm	Modification of the raw material with iron nitrate. Low or no influence of pH and temperature on adsorption.	Foletto et al. (2017) [55]
Nanoadsorbent from the fruit coat of a Kendu tree	AW	Tartrazine	125 min	7.9	6	70	PSO	Langmuir	Removal rate increased when the adsorbent dose and temperature were increased before reaching a plateau, but decreased when the initial dye concentration was increased.	Biswal et al. (2022) [6]
Powdered activated carbon from rubber seed and its shell	B	Methylene blue and Congo red	-	Methylene blue: 769.2 Congo red: 458.4	4 and 11	-	PSO	Congo red: Langmuir	Pollutant removal increased with increasing contact time, and decreased with increasing initial concentration, temperature and ionic strength.	M.Nizam et al. (2021) [57]
Calcite, zeolite, sand, and iron filings	R	NO_3_^−^, PO₄³⁻, and 6 metals	-	-	-	-	-	Freundlich	Most of the filter materials used had lower removal efficiency when pollutants were present simultaneously. Iron filings were found to be the most effective material for removal.	Reddy et al. (2014) [13]
Carnauba straw (CS) and cashew leaf (CF)	AW	Cu(II)	120 min	CS: 9.5 CL: 1.7	6	-	PSO	CS: Langmuir CL: Freundlich model	The decrease in particle size allowed the increase in the adsorption rate.	Pereira et al. (2021) [82]
Clay	R	Cu(II), Co(II), Ni(II), and Pb(II)	60 min	1.1	8	-	-	Freundlich and Langmuir	The adsorption capacity increased when the pH increased.	Es-sahbany et al. (2022) [7]
Co-system of strain L1 immobilized on peanut shell biochar (PSB)	B	Ni(II), Cr(VI), Cu(II), and NO_3_^-^	Heavy metals on PSB: 8h	Ni(II) on PSB: 24.7	5–8	-	Ni(II): PSO Cr(VI): Elovich Cu(II): PFO	Ni(II) on PSB: Langmuir:	A practical application of the system to remove pollutants was simulated in a sequential batch reactor. Adsorption increased with pH for Cu(II) and Ni(II), and decreased for Cr(VI) probably because it was reduced to Cr(III).	An et al. (2022) [16]
Flax fibers	AW	Cu(II), Pb(II), and Zn(II)	60 min	Cu(II): 7.8 Pb(II): 23.3 Zn(II): 4.6	6.4	-	Cu(II) and Pb(II): PSO Zn(II): PFO	Langmuir	A competition effect of pollutants for adsorption sites has been demonstrated. Lead was the most adsorbed metal in the single and ternary solutions.	Kajeiou et al. (2020) [85]
Flax fibers	AW	Cu(II), Pb(II), and Zn(II)	60 min	Cu(II): 9.9 Pb(II): 10.7 Zn(II): 8.4	4–7	-	PSO	Langmuir	The adsorption capacity increased when the amount of adsorbent increased.	Abbar et al. (2017) [84]
Hydrochloric acid treated peat and citric acid-treated sawdust	AW	Zn(II), Cr(III), Ni(II), and Cu(II)	15–30 min	Ni by hydrochloric acid treated peat: 21	-	-	-	-	Modification of the raw material with acids (hydrochloric for peat and citric for sawdust).	Gogoi et al. (2018) [79]
Lignocellulosic (flamboyant) biomass biochar	B	Pb(II), Hg(II), and Zn(II)	24h	0.024–0.411 mmol/g	-	40	-	Combinaison of statistical physics models and DFT calculations	Study of the adsorption process using statistical physics models and density functional theory calculations. Antagonistic adsorption for all heavy metals.	Sellaoui et al. (2019) [66]
Synthetic cancrinite	R	Cu(II) and Zn(II)	-	Single solution: 118.3 and 67.0 for Cu(II) and Zn(II)	-	50	PSO	Langmuir	Cancrinite was synthesized from crude muscovite via activation with sodium hydroxide and is more efficient for the removal of Cu(II). Adsorbed amount decreased from a single solution to a binary solution, showing a competition effect.	Selim et al. (2019) [63]
Bamboo-based biochar/montmorillonite composite	B	NO_3_^−^	100 min	Biochar: 5 Composite: 9	4	-	-	Langmuir	Nitrate removal was rapid (10 min), and then the adsorption rate gradually decreased with time.	Viglašová et al. (2018) [77]
Biochar from wheat straw	B	NO_3_^-^ and PO₄^3-^	-	NO_3_^−^: 2.5 PO₄³⁻: 16.6	NO_3_^−^: 3 PO₄³⁻: 6	-	-	Langmuir	Chloridic acid treatment of wheat straw resulted in a higher surface area and pore volume.	Li et al. (2014) [90]
Sugarcane Bagasse-derived biochar	B	NO_3_^-^	60 min	28.2	4.6	-	PSO	Langmuir	Modification of biochar with epichlorohydrin, N,N-dimethylformamide, ethylenediamine, and trimethylamine. By increasing the pH and introducing the nitrate in the presence of coexisting ions, the adsorption decreased. On the contrary, it increased by increasing the adsorbent dosage and the temperature.	Divband Hafshejani et al. (2016) [8]
Modified natural fabrics based on cotton (MC) and wool (MW)	AW	Pirimiphos-methyl and monocrotophos	2h	MC: 333.3–454.6 MW: 500.0–625.0	-	-	PSO	Langmuir	The fabrics were modified with the synthetic polymer polyethyleneimine, which increased the adsorption capacity of wood for both pesticides.	Abdelhameed, El-Zawahry and E. Emamc (2018) [80]
Nanoadsorbent from the fruit coat of a Kendu tree	AW	Tartrazine	125 min	7.9	6	70	PSO	Langmuir	Removal rate increased when the adsorbent dose and temperature were increased before reaching a plateau, but decreased when the initial dye concentration was increased.	Biswal et al. (2022) [6]
P-doped biochar from corn straw	B	6 pesticides	Atrazine: 20 min	Atrazine: 79.6	-	-	PSO	Freundlich	Activation with phosphoric acid resulted in improved adsorption performance of the biochar. Adsorption rates of the six pesticides increased with increasing adsorbent dosage.	Suo et al. (2019) [78]
Tangerine seed-derived biochar	B	Bendiocarb, metolcarb, isoprocarb, pirimicarb, carbaryl, and methiocarb	12 min	7.97–93.5	7	20	PSO	Langmuir	Increasing the carbonization temperature and time resulted in an increase in biochar pore width and pesticide removal efficiency, respectively.	Wang et al. (2020) [56]
Waste rubber tire-derived biochar	B	Methoxychlor, methyl parathion, and atrazine	60 min	88.9–112.0	2	25	PFO	Langmuir	A direct relationship was found between the adsorption capacity and the octanol–water partition coefficient values of the pollutants. By increasing the pH, the adsorption decreased.	Gupta et al. (2011) [59]
Biochar from algae	B	Ciprofloxacin	-	Brown algae-derived biochar: 250	7	25	PSO	Langmuir	Different types of products were generated during pyrolysis: aromatics, hydrocarbons, phenols, acids, alcohols, furans, nitrogenous chemicals.	Nguyen et al. (2022) [53]
Modified biomass of green alga *Scenedesmus obliquus*	A	Tramadol	45 min	140.2	7	-	Tramadol: PSO	Tramadol: Freundlich	Modification of the raw material with a sodium hydroxide solution that increased the removal. Competitive adsorption occurred between the pharmaceutical pollutants.	Ali et al. (2018) [64]
*Moringa oleifera* seed husk biomass	AW	Diclofenac	1080 min	28.7	5	-	PSO	Freundlich	Chemical modification of the raw material with methyl alcohol and nitric acid solution, followed by physical modification in a muffle for 1 h at 300 °C. Adsorption decreased with increasing pH.	Araujo et al. (2018) [11]
White-rot fungi (*Trametes versicolor* and *Ganoderma lucidum*)	MB	13 pharmaceutical pollutants	-	-	-		-	-	Individual and combined fungal bioassays were tested to produce a raw material for the production of biodiesel via the valorization of fungal sludge generated during the disposal process have been carried out.	Vasiliadou et al. (2016) [86]
Green tide algae *Ulva prolifera*	A	Phenanthrene	-	-	-	30	Two-stage PFO	-	An increase in nutrients, temperature, and initial pollutant concentration resulted in an increase in the rate of phenanthrene removal.	Zhang et al. (2017) [54]
Coconut shell activated carbon	B	Dioxins	-	600	-	-	-	-	Adsorption capacity determined according to linear relationships between gas properties and adsorption behaviors.	Guo et al. (2016) [91]
Processed montmorillonite clays	R	PCBs	-	-	-	26–37	-	Langmuir	Steric hindrance limited the access of the pollutant to the montmorillonite clay surfaces, reducing the adsorption capacity	Wang et al. (2019) [24]
Raw and modified plant residues	AW	Naphthalene, acenaphthene, phenanthrene, and pyrene	24–50h	-	-	-	PSO	Freundlich	Acid hydrolysis was used to modify the raw material. Sorption coefficients were negatively correlated with polarity and positively correlated with adsorbent aromaticity.	Xi and Chen (2014) [83]
Seagrass leaf powder	AW	Acenaphthylene (A), phenanthrene (P), and fluoranthene (F)	F: 6h P: 24h A: 120h	F: 2.2 P: 2.1 A: 1.1	-	-	PSO	Freundlich	Removal efficiency increased with increasing amount of adsorbent while maximum adsorption capacity decreased, presumably since saturation could not be reached due to increasing dosage.	Akinpelu et al. (2021) [10]
Wood waste-derived biochar	B	19 PAHs, 23 Nitro-PAHS, and 9 Oxygenated-PAHs	-	2.0	-	-	PSO	PAHs: Langmuir N-PAHs: category IV O-PAHs: category II	A molecular model was used to simulate the fundamental properties of the biochar. Destruction of micro-pores and formation of meso-pores in the biochar was observed following acid treatment.	Zhou et al. (2021) [92]

**Table 4:** Optimal parameters for biomass pyrolysis according to several studies.

Biomass	Gas Used	Gradient of Temperature (°C/min)	Maximum Temperature (°C)	Carbonization Time (min)	Chemical Activating Agent	Reference
Peanut shell	N_2_	10	500	120	-	An et al. (2022) [16]
Marine algae	N_2_	10	700	120	ZnCl_2_	Nguyen et al. (2022) [57]
Bamboo biomass	N_2_	-	460	120	-	Viglašová et al. (2018) [84]
[Litchi peels	N_2_	10	800	120		Foletto et al. (2017) [59]
Lignocellulosic biomass	N_2_	10	600	120	-	Sellaoui et al. (2019) [70]
Corn straw and corncob	-	-	300	120	H_3_PO_4_	Suo et al. (2019) [85]
Rubber seed and shell	-	-	800	480	H_2_SO_4_ (after the pyrolysis)	M.Nizam et al. (2021) [61]
Tangerine seed	-	10	600	240	H_3_PO_4_	Wang et al. (2020) [60]
Waste rubber tire	-	-	900	120	KOH	Gupta et al. (2011) [63]

## Data Availability

Not applicable.

## Abbreviations

AAS	Atomic absorption spectroscopy
GC-FID	Gas chromatography—flame ionization detector
GC–MS/MS	Gas chromatography—tandem mass spectrometry
HPLC	High-performance liquid chromatography
ICP-AES	Inductively coupled plasma atomic emission spectroscopy
ICP-MS	Inductively coupled plasma mass spectrometry
LC-MS/MS	Liquid chromatography—tandem mass spectrometry
PFO	Pseudo-first order model
PPCPs	Pharmaceuticals and personal care products
PSO	Pseudo-second order model
Py-GC/MS	Pyrolysis–gas chromatography–mass spectrometry
UHPLC	Ultra high-performance liquid chromatography
UV-Vis	UV–visible spectrophotometry
